# Supplementary material for: Patterns of Intron Gain and Loss in Fungi
Source: PLoS Biol. 2004 Nov 30;2(12):e422. doi: 10.1371/journal.pbio.0020422 (PMC532390; doi:10.1371/journal.pbio.0020422)
Supplement: Table S1 — Also available at http://genes.mit.edu/NielsenEtAl/. (4.3 MB ZIP). [file pbio.0020422.st001.zip › NielsenEtAl/html/1352.html]

AN0784.1.NCU03693.1.MG09218.1.FG01857.1


```
 CLUSTAL W (1.82) Multiple Sequence Alignments - Introns Inserted


Sequence 1: MG09218.1	284 aa
Sequence 2: FG01857.1	287 aa
Sequence 3: NCU03693.1	286 aa
Sequence 4: AN0784.1	281 aa
Alignment Length: 290 aa
Number Identitical Residues: 148 aa
Alignment Score (without introns) 7195


MG09218.1 	--MTEAANLESFPTLFSLKGKVAVVTGGSRGLGLSAASA~LLQAGCSKVFITSRKASACD
NCU03693.1	--MADAQ-LNDFPSLFSVKGKVAVVTGGSRGLGLSAASA2LLQAGASKVFISSRKASACD
FG01857.1 	--MAEAQ-LNDFPSLFSLKGKVAVVTGGSRGLGLHAASA2FLQAGASKVFISSRKASACE
AN0784.1  	MSASEQVQLNNFSSIFSLEGKVAVVTGGSRGLGLYAASG2LLQAGCSKVYITSRKASACE
          	 : ::  .*:.*.::**::*************** ***. :****.***:*:*******:

MG09218.1 	SACATLNALPGIS--GRAISVPSDSANEAGVKKLLELVSAHTDHVDILMANAGASWGAPF
NCU03693.1	SAVKTLNALPGLAPGAKAISIPADAATLSGVQHLVSEVAKHTDHVDILLANAGATWGAPF
FG01857.1 	EACKALNALPNLQPGAVAISVPADSAKFEGVESLLAQVKKHTDRVDILFANAGATWGESF
AN0784.1  	SAVAALNALPNKRPGAQAISIPADSSKVSEIERLVSEVSKTTSHIDILFANAGATWGEEF
          	.*  :*****.  ... ***:*:*::.   :: *:  *   *.::***:*****:**  *

MG09218.1 	DSHPDDAFAKVMDLNVRAVFNTVRLFAPLLQKRATIDDPSRVIITASVAGLGIGTLGKNG
NCU03693.1	DEHPDSAFAKVMDLNVRGVFNTIREFAPLLQKKASVEDPSRVIITASVAGLGIGTIGKQG
FG01857.1 	DTHPDSAFAKVMDLNVKAVFNTIRLFTPLLEKSATIQDPSRVLITASVAGLAVGTIGKQG
AN0784.1  	EKFPESAFDKVLDLNVKSVFFTIQKFFPLLSAKASLEDPSRVIITGSVAGITLGSLGKNG
          	: .*:.** **:****:.** *:: * ***.  *:::*****:**.****: :*::**:*

MG09218.1 	T0YGYSASKAAVLHLGNNLAVELGPRHITVNSIAPGFFPSKMANGLLEVSGGADNIAKAN
NCU03693.1	T~YGYSASKAAVIHLGRNLAVELGPRGITVNSICPGFFPTKMSNGLLDMSGGKESIAAAN
FG01857.1 	T~YGYSASKAAVLHLGRNLAMELGPRHITVNSICPGFFPSKMSNGLLEMSGGADQFAAAN
AN0784.1  	S~FSYSASKAAVLHMTRNLAVELGPKHVNVTAIAPGIYPSKMANGLIEIQGGMKEVEAAS
          	: :.********:*: .***:****: :.*.:*.**::*:**:***:::.** ...  *.

MG09218.1 	PMRRLGRPEDIAGVVVYLASRAGSHVNGACIAIDGGAMWQRGELMLEAKL--
NCU03693.1	PMKRLGRPEDIAGVVVYLASRAGAHVNGATIAIDGGAMWAKGQLNVESAKL-
FG01857.1 	PMRRLGQPEDIAGVVVYLASRAGAHVNGETVAIDGGALWQRGELMIAEKAKL
AN0784.1  	PNKRLGRPEDIAGLVVFLASRAAGHLNGSVITTDGGAHL-KGRM--------
          	* :***:******:**:*****..*:**  :: ****   :*.:
```
